# Activation of endothelial β-catenin signaling induces heart failure

**DOI:** 10.1038/srep25009

**Published:** 2016-05-05

**Authors:** Akito Nakagawa, Atsuhiko T. Naito, Tomokazu Sumida, Seitaro Nomura, Masato Shibamoto, Tomoaki Higo, Katsuki Okada, Taku Sakai, Akihito Hashimoto, Yuki Kuramoto, Toru Oka, Jong-Kook Lee, Mutsuo Harada, Kazutaka Ueda, Ichiro Shiojima, Florian P. Limbourg, Ralf H. Adams, Tetsuo Noda, Yasushi Sakata, Hiroshi Akazawa, Issei Komuro

**Affiliations:** 1 Department of Cardiovascular Medicine, Osaka University Graduate School of Medicine, Suita, Osaka 565-0871, Japan; 2 Department of Cardiovascular Medicine, Graduate School of Medicine, The University of Tokyo, Bunkyo-ku, Tokyo 113-8655, Japan; 3 Japan Agency for Medical Research and Development, AMED-CREST, Chiyoda-ku, Tokyo 100-0004, Japan; 4 Department of Cardiovascular Regenerative Medicine, Osaka University Graduate School of Medicine, Suita, Osaka 565-0871, Japan; 5 Department of Medicine II, Kansai Medical University, Hirakata, Osaka 573-1191, Japan; 6 Experimentelle Gefäßmedizin und Transplantationsforschung/ Koordinator Hypertoniezentrum, Klinik für Nieren- und Hochdruckerkrankungen, Medizinische Hochschule Hannover Carl-Neubergstr. 130625 Hannover, Germany; 7 Max Planck Institute for Molecular Biomedicine, Department of Tissue Morphogenesis, University of Münster, Faculty of Medicine, Münster, Germany; 8 Department of Cell Biology, The Cancer Institute, Japanese Foundation for Cancer Research, Koto-ku, Tokyo 135-8550, Japan

## Abstract

Activation of β-catenin-dependent canonical Wnt signaling in endothelial cells plays a key role in angiogenesis during development and ischemic diseases, however, other roles of Wnt/β-catenin signaling in endothelial cells remain poorly understood. Here, we report that sustained activation of β-catenin signaling in endothelial cells causes cardiac dysfunction through suppressing neuregulin-ErbB pathway in the heart. Conditional gain-of-function mutation of β-catenin, which activates Wnt/β-catenin signaling in *Bmx*-positive arterial endothelial cells (Bmx/CA mice) led to progressive cardiac dysfunction and 100% mortality at 40 weeks after tamoxifen treatment. Electron microscopic analysis revealed dilatation of T-tubules and degeneration of mitochondria in cardiomyocytes of Bmx/CA mice, which are similar to the changes observed in mice with decreased neuregulin-ErbB signaling. Endothelial expression of *Nrg1* and cardiac ErbB signaling were suppressed in Bmx/CA mice. The cardiac dysfunction of Bmx/CA mice was ameliorated by administration of recombinant neuregulin protein. These results collectively suggest that sustained activation of Wnt/β-catenin signaling in endothelial cells might be a cause of heart failure through suppressing neuregulin-ErbB signaling, and that the Wnt/β-catenin/NRG axis in cardiac endothelial cells might become a therapeutic target for heart failure.

Coronary blood flow is important for maintaining cardiac function, and neovascular formation is necessary to compensate for the increased oxygen demand of hypertrophic heart as well as developmental heart^1^. Endothelial cells (ECs) play critical roles in forming new vessels as well as regulating vessel functions, and recent findings suggest that ECs also protect the heart by secreting a variety of biologically active substances^2^,^3^. Neuregulin is one of such substances that is secreted from ECs and plays a cardioprotective role through activating ErbB signaling in cardiomyocytes^4^,^5^,^6^. Endothelial function is reported to be impaired in the failing heart and its dysfunction may cause heart failure^7^, however, the mechanisms of how endothelial function is impaired in the failing heart and how endothelial dysfunction induces heart failure are unknown.

Wnt/β-catenin signaling regulates various biological processes during embryonic development^8^. Wnt/β-catenin signaling also plays essential roles in maintaining tissue homeostasis and aberrant activation of Wnt/β-catenin signaling is involved in the pathogenesis of many diseases^8^,^9^,^10^. β-catenin protein is the key mediator of canonical Wnt signaling. In the absence of Wnt protein, cytosolic β-catenin is phosphorylated and degraded via proteasome pathway. Binding of Wnt protein to its receptors blocks the phosphorylation of β-catenin and increases its cytosolic level. Cytosolic β-catenin in turn translocates to the nucleus and regulate Tcf/Lef mediated gene expression^10^,^11^. Third exon of the β-catenin gene encodes the N-terminal part of the protein that contains phosphorylation/ubiquitination site responsible for its proteasomal degradation. Knock-in mice with LoxP sequence flanking exon 3 of β-catenin gene (*Ctnnb1*^*lox(ex3)/lox(ex3)*^ mice) generates β-catenin protein lacking its exon 3 (β-catenin (Δex3)) after Cre-mediated recombination and is widely used as a mice model for conditional activation of Wnt/β-catenin signaling together with cell type-specific Cre mice^12^. Previous reports used *Ctnnb1*^*lox(ex3)/lox(ex3)*^ mice to investigate the roles of endothelial Wnt/β-catenin signaling in angiogenesis during embryonic development and ischemia. Sustained activation of Wnt/β-catenin signaling in ECs blocks vascular remodeling in early embryonic development^13^ whereas activation of Wnt/β-catenin signaling in ECs promotes angiogenesis after myocardial infarction^14^. However, the role of endothelial Wnt/β-catenin signaling in the biological process other than angiogenesis is poorly understood.

We here demonstrate the novel role of endothelial Wnt/β-catenin signaling in the heart. Using a transgenic mouse model with tamoxifen (TAM)-inducible, endothelial-specific expression of β-catenin (Δex3), we found that sustained activation of β-catenin signaling in ECs impairs cardiac function leading to severe heart failure. Mechanistically, activation of endothelial β-catenin signaling suppressed the expression of neuregulin (NRG), resulting in reduced activity of ErbB signaling of cardiomyocytes. Administration of recombinant NRG1 ameliorated ErbB signaling and cardiac function of the transgenic mice. These results suggest that sustained activation of β-catenin signaling in ECs causes heart failure in a NRG-ErbB signaling dependent manner.

## Results

### Inducible activation of Wnt/β-catenin signaling in arterial ECs

**Figure 1:**
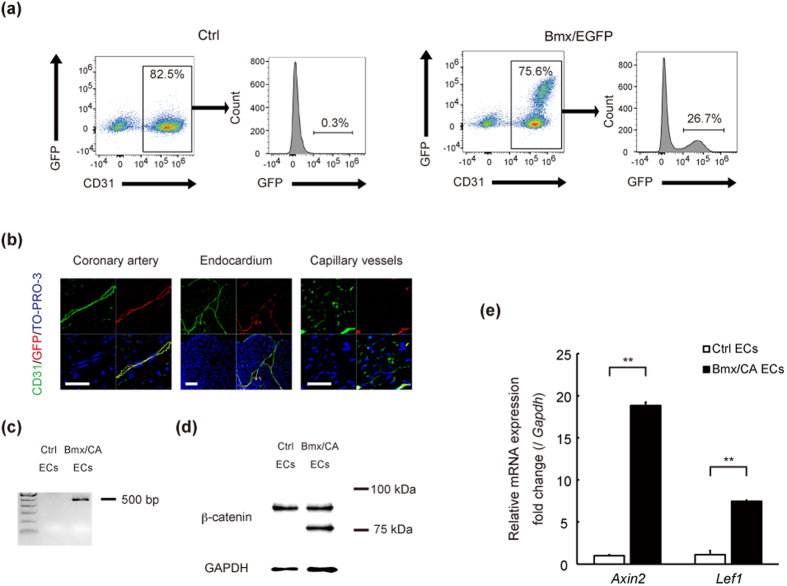
Inducible activation of Wnt/β-catenin signaling in arterial ECs. (**a**) Flow cytometric analysis of ECs. ECs were collected from the heart of *Bmx-CreER*^*T2*^ mice (Ctrl) or from *Bmx-CreER*^*T2*^ crossed with *CAG-CAT-EGFP* mice (Bmx/EGFP) 1 week after the TAM treatment. (**b**) Immunofluorescent staining of cardiac tissue for GFP (red), CD31 (green), and TO-PRO-3 (blue). Scale bars: 20 μm. (**c, d, e**) Genotyping PCR (**c**), western blot (**d**) and quantitative RT-PCR analysis (**e**) of cardiac ECs isolated from Ctrl (*Bmx-CreER*^*T2*^ with *Ctnnb1*^*+/+*^) mice (Ctrl ECs) and Bmx/CA mice (Bmx/CA ECs). (**c**) Floxed allelle of β-catenin (=500 bp) was detected in Bmx/CA ECs but not in Ctrl ECs. (**d**) β-catenin protein lacking exon3 (=75 kDa) was detected in Bmx/CA ECs but not in Ctrl ECs. (**e**) Expression levels of Wnt/β-catenin signaling target genes (*Axin2* and *Lef1*) were higher in Bmx/CA ECs compared with Ctrl ECs. **P < *0.01*.

A previous report crossed *Tie2-Cre* or *VE-cadherin-Cre* mice with *Ctnnb1*^*lox(ex3)/lox(ex3)*^ mice to induce EC-specific expression of β-catenin (Δex3) but these mice were embryonic lethal^13^. We therefore used *Bmx-CreER*^*T2*^ transgenic mice to achieve inducible expression of degradation-resistant β-catenin (Δex3) in an arterial EC-specific manner and to investigate the role of β-catenin signaling in ECs of adult mice. *Bmx* is a member of the Tec tyrosine kinase gene family and is highly expressed in the ECs of arteries and in the endocardium, but not in the venular endothelium^15^. We first crossed *Bmx-CreER*^*T2*^ mice with enhanced green fluorescent protein reporter mice (*CAG-CAT-EGFP* mice)^16^, and confirmed that all the EGFP-positive cells were also CD31-positive and approximately a quarter of CD31-positive cells were EGFP-positive in the heart (Fig. 1a). Immunofluorescence analysis also showed that EGFP was expressed in the endothelium of coronary arteries and endocardium, but not in the capillary vessels (Fig. 1b). We then crossed *Bmx-CreER*^*T2*^ mice with *Ctnnb1*^*lox(ex3)/lox(ex3)*^ mice (Bmx/CA mice) to observe the effect of conditional activation of β-catenin signaling in endothelial cells. Floxed *Ctnnb1* allele and β-catenin protein lacking exon3 were detected in cardiac ECs of Bmx/CA mice but not of control *Bmx-CreER*^*T2*^ *Ctnnb1*^+/+^ (Ctrl) mice (Fig. 1c,d). β-catenin protein lacking exon3 were not detected in cardiomyocytes of Bmx/CA mice (Supplemental Fig. S1) suggesting that Cre-mediated recombination did not directly affected cardiomyocytes. We also observed the up-regulation of Wnt/β-catenin target genes such as *Axin2* and *Lef1* in cardiac ECs of Bmx/CA mice (Fig. 1e), indicating that β-catenin (Δex3) protein in ECs are functional and activates Wnt/β-catenin signaling in Bmx/CA mice.

### Activation of β-catenin signaling in ECs causes heart failure

**Figure 2:**
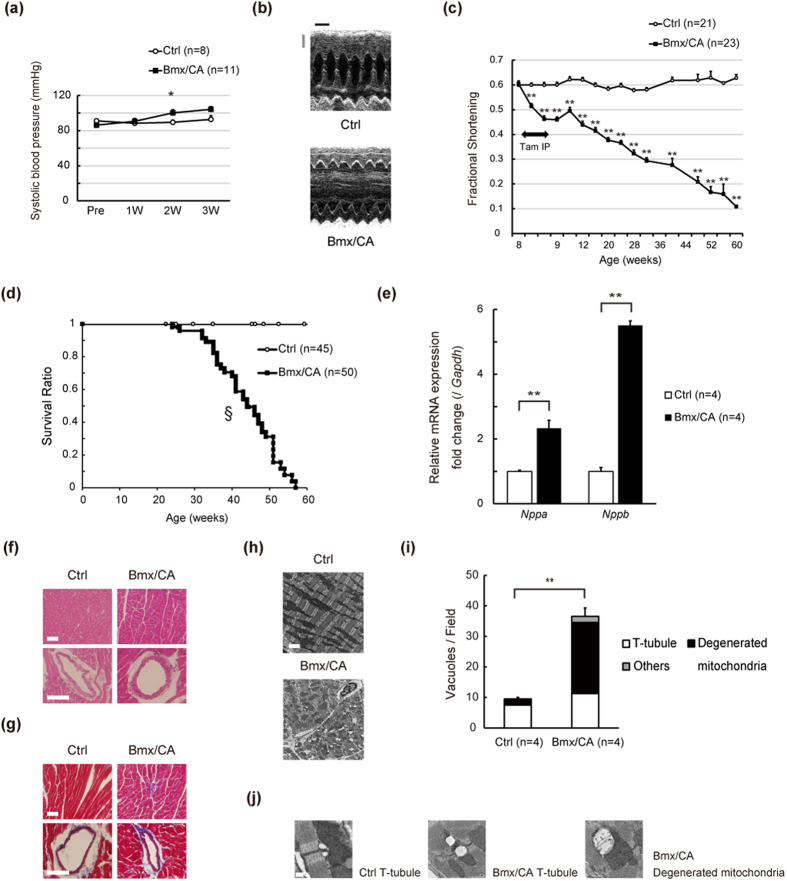
Activation of β-catenin signaling in ECs causes heart failure. (**a**) Systolic blood pressure was examined before (Pre) and 1, 2, and 3 weeks after TAM treatment. *P < *0.05* versus Ctrl (*n* = 8–11). (**b**) Representative M-mode echocardiogram at 6 months after TAM treatment. Scale bars: 1 mm (gray), 100 msec (black). (**c**) Cardiac function of the mice was evaluated by echocardiography before, 3^rd^ and 5^th^ day of TAM treatment, and at the indicated time point. **P < *0.01* versus Ctrl. (**d**) Kaplan-Meier curve showing survival rate of Ctrl and Bmx/CA mice after TAM treatment. ^§^*P* < 0.001 versus Ctrl. (**e**) Quantitative RT-PCR analysis of the heart failure related genes. **P < *0.01* versus Ctrl. (**f**) Hematoxylin and eosin staining and (**g**) Masson and Trichrome’s staining of heart tissue from Ctrl and Bmx/CA mice at 1 year after TAM. Scale bars: 50 μm. (**h**) Representative electron microscopic images of heart tissue from Ctrl and Bmx/CA mice at 1 year after TAM. Scale bar: 2.5 μm. (**i**) Quantitation of the vacuoles. Vacuoles with more than 0.25 μm diameters were counted in 12 randomly selected fields (18.75 × 18.75 μm^2^). “T-tubule” was defined as typical circular vacuoles nearby Z-bands. “Degenerated mitochondria” was defined as dilated vacuoles within the mitochondria. Undefined vacuoles were defined as “others”. **P < *0.01* versus Ctrl. (**j**) Representative images of the “T-tubule” and “Degenerated mitochondria”. Scale bar: 0.25 μm.

Since ECs play critical roles in regulation of vascular functions, we first analyzed the blood pressure of Bmx/CA mice. Blood pressure of Bmx/CA mice was comparable to that of Ctrl mice (Fig. 2a). In clear contrast, cardiac function was progressively impaired in Bmx/CA mice compared with Ctrl mice (Fig. 2b,c, Supplementary Table 1), resulting in nearly 100% mortality at 60 weeks of age (50 weeks after TAM treatment) (Fig. 2d). The heart weight/body weight ratio was significantly higher in Bmx/CA mice (Supplementary Fig. S2a), and expression levels of genes related to heart failure such as *Nppa* and *Nppb* as well as fibrosis marker gene *Col1a1* were also higher in the heart of Bmx/CA mice compared with Ctrl mice (Fig. 2e, Supplementary Fig. S2b). TUNEL staining revealed that the number of apoptotic cardiomyocytes was not increased in the heart of Bmx/CA mice (Supplementary Fig. S2c). Capillary density was also comparable between Ctrl and Bmx/CA mice (Supplementary Fig. S2d) and no signs of tissue hypoxia was observed in the heart of Bmx/CA mice (Supplementary Fig. S2e). These results suggest that sustained activation of β-catenin signaling in ECs induces fatal heart failure without the involvement of cardiomyocyte death or cardiac ischemia. Gross histological analysis revealed that there was neither inflammatory nor fibrotic changes in the heart of Bmx/CA mice (Fig. 2f,g), however, electron microscopic analysis revealed characteristic changes such as dilatation of T-tubules and degeneration of mitochondria in the cardiomyocytes of Bmx/CA mice (Fig. 2h–j). These changes are similar to the changes in hearts of cardiomyocyte-specific ErbB2 receptor^17^,^18^ and ErbB4 receptor knockout mice^19^ and of the patients with trastuzumab-induced^20^ cardiomyopathy, suggesting that ErbB signaling might be involved in the cardiac phenotype of Bmx/CA mice.

### Endothelial Nrg1 expression and cardiac ErbB signaling are suppressed in Bmx/CA mice

**Figure 3:**
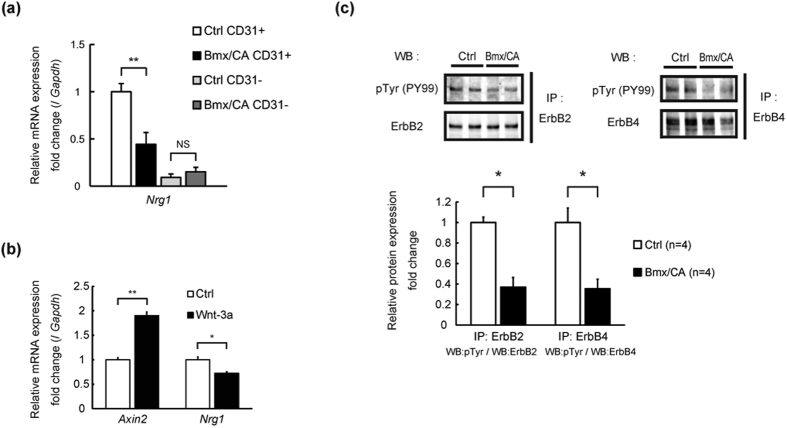
Endothelial *Nrg1* expression and cardiac ErbB signaling are suppressed in Bmx/CA mice. (**a**) Quantitative RT-PCR analysis of *Nrg1* expression in cardiac ECs isolated from Bmx/CA mice (Bmx/CA ECs) compared with ECs isolated from Ctrl mice (Ctrl ECs). **P < *0.01* (n = 4). (**b**) Quantitative RT-PCR analysis of *Nrg1* and *Axin2* expression in HCAECs stimulated with recombinant human Wnt3a (200 ng/mL) (Wnt-3a) or PBS containing 0.1% BSA (Ctrl). **P < *0.01*, *P < *0.05* (*n* = 4). (**c**) Immunoprecipitation and western blotting analysis of ErbB2 and ErbB4 phosphorylation. Lysates from the heart of Ctrl or Bmx/CA mice at 1 week after TAM were precipitated with anti-ErbB2 or anti-ErbB4 antibody and the precipitates were blotted with anti-phosphorylated tyrosine antibody (pTyr (PY99)) or with the antibody used for precipitation. Phosphorylation levels were analyzed from the band density using ImageJ (Bottom panel). *P < *0.05* (*n* = 4).

NRG1 is produced mainly by ECs^21^ and exerts cardioprotective effects through binding to ErbB2/ErbB4 receptors^4^,^5^,^6^. Mice deficient for either NRG1^22^, ErbB2^23^, or ErbB4^24^ showed embryonic lethality due to absence of trabeculae formation in the ventricle. Cardiomyocyte-specific deletion of either ErbB2^17^,^18^ or ErbB4^19^ resulted in dilated cardiomyopathy-like phenotype, e.g. cardiac dysfunction and increased expression of heart failure marker genes, with abnormal electron microscopic finding such as vacuole formation and dilation of T-tubules. We therefore hypothesized that reduced NRG-ErbB signaling might be involved in the mechanism of heart failure in Bmx/CA mice. Expression levels of *Nrg1* were significantly lower in cardiac ECs prepared from Bmx/CA mice compared with Ctrl mice. Expression levels of *Nrg1* in non-endothelial cells were lower compared with endothelial cells and were comparable between Ctrl and Bmx/CA mice (Fig. 3a). Recombinant human Wnt3a increased the expression of *Axin2* whereas it suppressed the expression of *Nrg1* in cultured human coronary arterial endothelial cells (HCAECs) (Fig. 3b), suggesting that activation of Wnt/β-catenin signaling is tightly associated with decreased *Nrg1* expression in endothelial cells. Moreover, phosphorylation levels of ErbB2 and ErbB4 proteins were lower in the heart of Bmx/CA mice as compared with Ctrl mice (Fig. 3c). These results indicate that activation of β-catenin signaling in ECs suppressed the activity of cardiac ErbB signaling through down-regulating the expression of *Nrg1* in ECs.

### Administration of NRG1 protein rescued the cardiac phenotype of Bmx/CA mice

**Figure 4:**
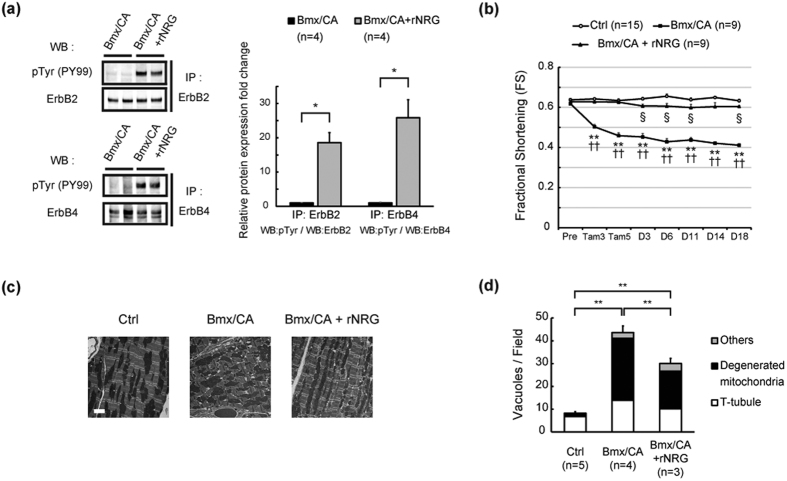
Administration of NRG1 rescued the cardiac phenotypes of Bmx/CA mice. (**a**) Immunoprecipitation and western blotting analyses of ErbB2 and ErbB4 phosphorylation. Lysates from the heart of Bmx/CA treated with or without rNRG were precipitated with anti-ErbB2 or anti-ErbB4 antibody and the precipitates were blotted with anti-phosphorylated tyrosine antibody (pTyr (PY99)) or with the antibody used for precipitation. Phosphorylation levels were analyzed from the band density using ImageJ (Right panel). *P < *0.05* (*n* = 4). (**b**) Cardiac function of Ctrl mice (Ctrl) and Bmx/CA mice treated with vehicle (Bmx/CA) or rNRG (Bmx/CA + rNRG) were evaluated by echocardiography before (Pre), 3^rd^ and 5^th^ day of TAM treatment (Tam3 and Tam5), and at the indicated time point after the 5 day- TAM treatment. **P < *0.01* versus Ctrl, ^††^*P* < *0.01* versus Bmx/CA + rNRG, ^§^*P* < *0.05* versus Ctrl. (**c**) Representative electron microscopic images of the heart tissue from Ctrl and Bmx/CA mice treated with vehicle (Bmx/CA) or rNRG (Bmx/CA + rNRG). Scale bar: 2.5 μm. (**d**) Quantitation of vacuole formations. Vacuoles were evaluated as described in Fig. 2i. **P < *0.01*.

We next examined whether decreased *Nrg1* expression in endothelial cells and reduced neuregulin/ErbB signaling are involved in the mechanism of cardiac dysfunction in Bmx/CA mice. Administration of recombinant NRG1 recovered the phosphorylation of cardiac ErbB2 and ErbB4 receptors (Fig. 4a). Cardiac function of Bmx/CA mice was also improved after administration of NRG1 (Fig. 4b, Supplementary Table 2.), which was accompanied by significant improvement of electron microscopic findings, i.e., decreased number of dilated T-tubules and degenerated mitochondria in cardiomyocytes (Fig. 4c,d). These results suggest that decreased expression of *Nrg1* and activity of ErbB signaling were responsible for cardiac dysfunction of Bmx/CA mice.

## Discussion

Wnt/β-catenin signaling plays an important role in cardiac hypertrophy and ischemic injury^25^,^26^,^27^, however, little is known about the role of Wnt/β-catenin signaling in heart failure. In this study, we showed that sustained activation of β-catenin signaling in ECs induces heart failure in adult mice. Activation of β-catenin signaling in ECs decreased the expression levels of endothelial *Nrg1* and suppressed the activity of cardiac ErbB signaling. Administration of NRG protein rescued the cardiac dysfunction that is caused by activation of β-catenin signaling. These results collectively suggest that activation of Wnt/β-catenin signaling in cardiac ECs suppresses cardiac NRG-ErbB signaling, resulting in development of heart failure.

As Wnt/β-catenin signaling in ECs regulates angiogenesis during embryogenesis^13^, we first postulated that sustained activation of endothelial Wnt/β-catenin signaling impairs the angiogenesis in the heart, thereby inducing hypoxia. However, there were no significant differences in vascular density (Supplementary Fig. S2d), and in myocardial hypoxia between Bmx/CA and Ctrl mice (Supplementary Fig. S2e), indicating that angiogenesis or tissue hypoxia were less likely to be the cause of cardiac dysfunction in Bmx/CA mice.

Angiotensin II or phenylephrine treatment has been reported to decrease the expression of NRGs in ECs *in vitro*^28^, however, it is not known how endothelial NRG production is regulated *in vivo*. In the present study, we showed that activation of β-catenin signaling suppressed NRG1 production from ECs both *in vitro* and *in vivo*. Whether activation of β-catenin signaling suppress the expression of *Nrg1* gene through Tcf-dependent canonical Wnt signaling or through interaction with other signaling cascade such as Foxo signaling^29^, Notch signaling^30^, or Hippo signaling^31^ require further investigations.

We observed T-tubule dilatation and mitochondria degeneration that appear like “vacuoles” through electron microscopic analysis of Bmx/CA mice. Vacuole-like structure formation in the cardiomyocytes is also observed in the heart of ErbB knockout mice^32^, doxorubicin-induced heart failure model mice^33^,^34^ and in the patients with trastuzumab (Herceptin)-induced cardiomyopathy^20^, suggesting that activation of β-catenin or Wnt/β-catenin signaling in endothelial cells might be involved in the molecular mechanism of those diseases. However, as *Ctnnb1*^*lox(ex3)/lox(ex3)*^mice might increase the cytosolic β-catenin and induce/suppress the expression of Wnt target genes to the supraphysiological level, whether there are relevant human cardiac diseases that are related with high level of β-catenin or Wnt/β-catenin signaling in endothelial cells remains to be elucidated in the future study.

**Figure 5:**
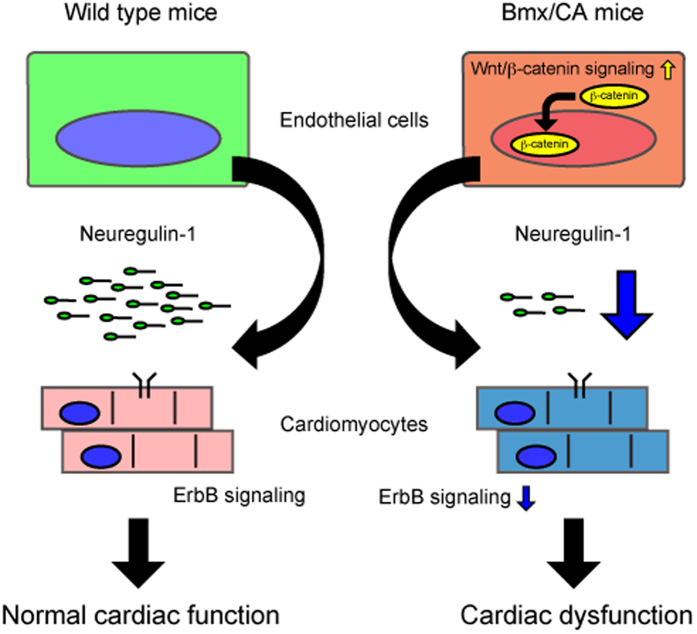
Sustained endothelial Wnt/β-catenin signaling and heart failure. Expression of degradation-resistant β-catenin (Δex3) activated Wnt/β-catenin signaling and suppressed the expression/secretion of neuregulin-1 in/from endothelial cells. Low neuregulin level suppressed cardiac ErbB signaling and promoted cardiac dysfunction.

Dysfunction of the cardiomyocytes plays a central role in the pathophysiology of heart failure. There are many “heart failure model mice” with cardiomyocyte specific gene modification^35^. Recent reports, however, highlight the role of non-cardiomyocytes in the heart, e.g. endothelial cells, fibroblasts and blood cells, in maintaining ventricular homeostasis and proper cardiac function^1^,^36^. In the present study, heart failure was observed in the mice with endothelial cell specific gene modification, suggesting that dysfunction of endothelial cells may play a primary role in the pathogenesis of heart failure (Fig. 5). Approaches targeting the signaling cascades in non-cardiomyocytes, e.g. Wnt/β-catenin signaling in endothelial cells, might become a novel therapeutic target for heart failure.

## Methods

### Reagents

Tamoxifen (T5648) was purchased from Sigma-Aldrich. Recombinant human NRG1-β1/HRG1-β1 (396-HB) and recombinant human Wnt3a (5036-WN) were from R&D Systems. Mouse monoclonal antibody against β-catenin (610154) and rat monoclonal antibody against CD31 (PECAM1) (553370) were from BD Biosciences. Rabbit polyclonal antibody against GFP (ab6556) was from Abcam. TO-PRO^®^-3 Iodide (642/661) (T3605) was from Invitrogen. Rabbit polyclonal antibody against ErbB2 (Neu) (C-18) (sc-284), rabbit polyclonal antibody against ErbB4 (C-18) (sc-283) and mouse monoclonal antibody against phosphorylated tyrosine (PY99) (sc-7020) were from Santa Cruz Biotechnology. Rabbit monoclonal antibody against GAPDH (14C10) (#2118) was from Cell Signaling Technology. Immunoprecipitation beads (Protein A Sepharose 4 Fast Flow (17-5280)) was from GE-Healthcare. Magnetic-activated cell sorting (MACS) beads, anti-rat IgG microbeads (130-048), were from Miltenyi Biotec. Secondary antibodies conjugated to Alexa Fluor 488 and Alexa Fluor 594 were from Molecular Probes. Hypoxyprobe^TM^-1 Omni Kit (HP3-100) was from Hypoxyprobe Inc.

### Animal model

We used 8- to 10-week-old male mice for tamoxifen administration. C57BL/6 mice were purchased from CLEA Japan. *Bmx-CreER*^*T2*^ mice^37^ and conditional β-catenin constitutive-activated mice (*Ctnnb1*^lox(ex3)/lox(ex3)^ mice)^38^ were crossed to generate EC-specific, tamoxifen (TAM)-inducible β-catenin stabilized mice (Bmx/CA mice). EGFP reporter mice (*CAG-CAT-EGFP*)^16^ were kindly gifted from J. Miyazaki, Osaka University. All mice backcrossed into C57BL/6 background were used for experiments. To induce Cre/loxP-mediated recombination in *Bmx-CreER*^*T2*^ mice, TAM dissolved in corn oil was injected intraperitoneally for 5 consecutive days (1 mg/50 μL/day). Blood pressure was measured in conscious mice by the tail-cuff system using with BP98A (Softron) according to manufacturer’s protocol. Left ventricular size and function of conscious mice were assessed by echocardiography using with the Vevo770 system (VisualSonics) with a 40MHz probe. Human recombinant NRG1-β1 was dissolved in phosphate buffer saline (PBS) containing 0.1% bovine serum albumin (BSA) and administrated intraperitoneally for 25 consecutive days (2.5 μg/day). Pimonidazole hydrochloride was dissolved in normal saline and administrated intraperitoneally 30 minutes before sampling (0.6 mg/kg). All animals were maintained in a virus-free facility on a 12-h light/12-h dark cycle and fed a standard diet. All experiments were approved by the Osaka University Institutional Review Board and performed under the guidelines of the Osaka University Committee.

### Genotyping of the animals

The PCR primers used for detection of floxed *Ctnnb1* allele were: 5′-TGAAGCTCAGCGCACAGCTGCTGTG-3′, 5′-GGACCCTCTGAGCCCTAGTCATTGC-3′

### Cell culture

Human coronary arterial endothelial cells (HCAECs) were purchased from Lonza (CLCC-2585). HCAECs were cultured in EGM^TM^-2MV BulletKit^TM^ (Lonza: CC-3162). HCAECs were seeded at 5 × 10^4^ cells per 0.33 cm^2^ (24-well plate). HCAECs were incubated at 37 °C and 5% CO_2_ for 24 hours and then treated with recombinant human Wnt3a (200 ng/mL, dissolved in PBS containing 0.1% BSA) for 24 hours.

### RNA analysis

Total RNA was extracted using with PureLink^®^ RNA mini or micro kit (Life Technologies: 12183020 or 12183016) according to the manufacturer’s instructions. RNA was treated with DNase and reverse-transcribed using QuantiTect Reverse Transcription Kit (QIAGEN: 205311). Quantitative real-time PCR (qRT-PCR) was performed using with Universal Probe Library (UPL) (Roche) and Light Cycler^®^ 480 Probes Master (Roche: 04887301001). Relative gene expression was normalized to the *Gapdh* gene expression using the comparative Ct method. Primer sequences and the corresponding UPL numbers were designed using online program provided by Roche (Supplementary Table 3).

### Protein analysis

Protein samples for western blotting were prepared using lysis buffer containing 20 mM HEPES (pH: 7.9), 150 mM NaCl, 5 mM EDTA, 15% glycerol, 1% TritonX-100, protease inhibitor cocktail (Roche: 11697498001), and phosphatase inhibitor cocktail (Roche: 04906837001). For immunoprecipitation experiments, heart tissue was minced and lysed in buffer containing 20 mM Tris-HCl (pH: 7.4), 150 mM NaCl, 1% NP-40, 3 mM EDTA, and protease/phosphatase inhibitors. Protein concentration was measured by BCA method. Densitometric analysis of the image was performed using ImageJ.

### Histological analysis

For morphological analysis, heart tissues were fixed with neutralized formaldehyde and embedded in paraffin. For fluorescent immunostaining, 5 μm-thick fresh frozen sections were stained and the nuclei were counterstained with TO-PRO-3. TUNEL staining was performed with TUNEL Apoptosis Detection Kit (Millipore: 17–141). Images were acquired by LSM700 confocal microscope (Zeiss) or FSX100 (Olympus). For electron microscope analysis, heart tissues were fixed with 2.5% glutaraldehyde solution (Wako: 072-02262). Septum and posterior free wall were dissected to 1–2 mm cube and ultrathin section was made using Reichert Ultracut S (Leica). Images were acquired with H-7650 (Hitachi).

### Cell isolation

Left ventricular tissue was minced and digested in digestion solution containing dispase II (Wako: 383–02281) (50 μg/mL) and collagenase type 1 (Wako: 035–17604) (1 mg/mL). Digests were further dissociated with 18 G needle. Remaining debris was removed and the supernatant was filtered through 40-μm cell strainer. Cells were suspended in PBS containing 4% FBS and incubated with rat monoclonal antibody against CD31 (PECAM1) (BD Biosciences: 553370). After washing with PBS with 0.5% BSA and 2 mM EDTA, cells were incubated with anti-rat IgG microbeads (Miltenyi Biotec: 130–048), and separated with separation columns (Miltenyi Biotec: 130-042-201). For flow cytometric analysis, cells were stained with LIVE/DEAD Fixable Aqua Dead Cell Stain Kit (Invitrogen: L34957), Tru Stain fcX (BioLegend 101320), and PE anti-mouse CD31 (BioLegend: 102408). After washing, cells were analyzed using Attune^®^ NxT acoustic focusing cytometer (Life Technologies). The data were analyzed by Flo Jo software (Tree Star). Isolation of adult cardiomyocytes using langendorff perfusion apparatus was performed as previously reported^39^.

### Statistical analysis

All data are presented as mean ± s.e.m. Statistical analysis was performed using Excel2013 (Microsoft, USA) with the add-in software Statcel3 (OMS, Japan). All variables were tested for distribution normality using Chi square test. When the data do not fit normal distribution in a group or normal variance between groups, non-parametric tests are used. In case of analyzing two groups, statistical difference was determined by the unpaired two-sided Student’s *t*-test (parametric) or the unpaired two-sided Mann-Whitney *U*-test (non-parametric). In case of analyzing multiple groups, the statistical difference was determined by Steel-Dwass test. Cumulative survival data was evaluated by Kaplan-Meier non-parametric regression analysis and the log-rank test. Significant differences were defined as *P* < 0.05.

## Additional Information

**How to cite this article**: Nakagawa, A. *et al*. Activation of endothelial β-catenin signaling induces heart failure. *Sci. Rep*. **6**, 25009; doi: 10.1038/srep25009 (2016).

## Supplementary Material

Supplementary Information
