# Author Correction: Activation of endothelial β-catenin signaling induces heart failure

**DOI:** 10.1038/s41598-018-34062-x

**Published:** 2018-10-29

**Authors:** Akito Nakagawa, Atsuhiko T. Naito, Tomokazu Sumida, Seitaro Nomura, Masato Shibamoto, Tomoaki Higo, Katsuki Okada, Taku Sakai, Akihito Hashimoto, Yuki Kuramoto, Toru Oka, Jong-Kook Lee, Mutsuo Harada, Kazutaka Ueda, Ichiro Shiojima, Florian P. Limbourg, Ralf H. Adams, Tetsuo Noda, Yasushi Sakata, Hiroshi Akazawa, Issei Komuro

**Affiliations:** 1 Department of Cardiovascular Medicine, Osaka University Graduate School of Medicine, Suita, Osaka, 565-0871 Japan; 2 Department of Cardiovascular Medicine, Graduate School of Medicine, The University of Tokyo, Bunkyo-ku, Tokyo 113-8655 Japan; 3 Japan Agency for Medical Research and Development, AMED-CREST, Chiyoda-ku, Tokyo 100-0004 Japan; 4 Department of Cardiovascular Regenerative Medicine, Osaka University Graduate School of Medicine, Suita, Osaka 565-0871 Japan; 5 Department of Medicine II, Kansai Medical University, Hirakata, Osaka 573-1191 Japan; 6 Experimentelle Gefäßmedizin und Transplantationsforschung/ Koordinator Hypertoniezentrum, Klinik für Nieren- und Hochdruckerkrankungen, Medizinische Hochschule Hannover Carl-Neubergstr, 130625 Hannover, Germany; 7 Max Planck Institute for Molecular Biomedicine, Department of Tissue Morphogenesis, University of Münster, Faculty of Medicine, Münster, Germany; 8 Department of Cell Biology, The Cancer Institute, Japanese Foundation for Cancer Research, Koto-ku, Tokyo 135-8550 Japan

Correction to: *Scientific Reports* 10.1038/srep25009, published online 05 May 2016

This Article contains incorrect echocardiographic data for Bmx/CA mice at 56w, which should be removed in Figure 2c and Supplementary Table 1. In addition, the LVFS values for 40w, 48w, 52w, 56w and 60w in Supplementary Table 1 are duplicates of the LVFS values for 16w, 20w, 24w, 28w and 32w, respectively. The correct Figure 2c is presented below as Figure 1 and the correct Supplementary Table S1 is linked to this Article.

**Figure 1:**
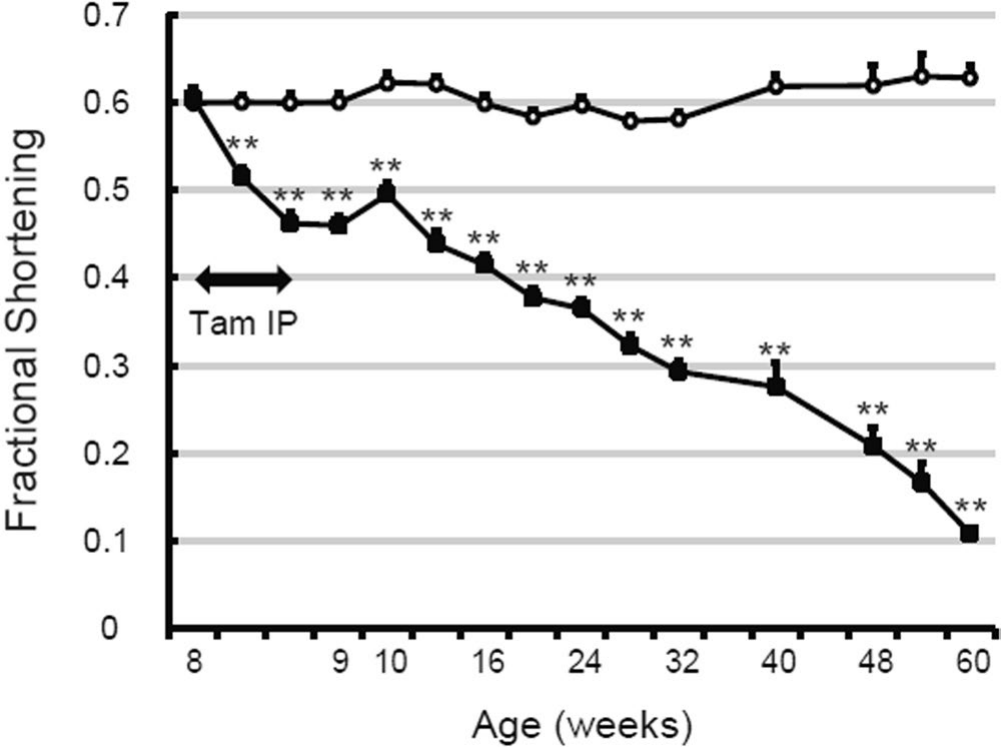
Activation of β-catenin signaling in ECs causes heart failure. (**c**) Cardiac function of the mice was evaluated by echocardiography before, 3^rd^ and 5^th^ day of TAM treatment, and at the indicated time point. **P < *0.01* versus Ctrl.

## Electronic supplementary material

Supplementary Table S1

