# Supplementary material for: Activation of endothelial β-catenin signaling induces heart failure
Source: Sci Rep. 2016 May 5;6:25009. doi: 10.1038/srep25009 (PMC4857119; doi:10.1038/srep25009)
Supplement: Supplementary Information [file srep25009-s1.pdf]

Supplementary Information for SREP-15-36660A

Activation of endothelial β-catenin signaling induces heart failure.

Akito Nakagawa, Atsuhiko T. Naito, Tomokazu Sumida, Seitaro Nomura, Masato Shibamoto, Tomoaki Higo, Katsuki Okada, Taku Sakai, Akihito Hashimoto, Yuki Kuramoto, Toru Oka, Jong-Kook Lee, Mutsuo Harada, Kazutaka Ueda, Ichiro Shiojima, Florian P. Limbourg, Ralf H Adams, Tetsuo Noda, Yasushi Sakata, Hiroshi Akazawa, & Issei Komuro

Supplemental Table 1., Nakagawa et al.

Echocardiographic parameters of Ctrl and Bmx/CA mice after tamoxifen treatment.

		LVFS			LVDd (mm)			HR (bpm)		
		mean	SEM	p-value	mean	SEM	p-value	mean	SEM	p-value
pre	Ctrl (n=21)	0.599	0.013	0.698	2.964	0.032	0.084	666.52	11.65	< 0.01
	Bmx/CA (n=23)	0.604	0.012		3.041	0.029		641.17	7.87	
8w (Tam3)	Ctrl (n=21)	0.600	0.008	< 0.01	2.920	0.038	< 0.01	674.00	6.44	0.860
	Bmx/CA (n=23)	0.514	0.011		3.110	0.031		670.13	7.11	
8w (Tam5)	Ctrl (n=21)	0.599	0.010	< 0.01	3.026	0.039	< 0.01	647.81	7.37	0.805
	Bmx/CA (n=23)	0.462	0.013		3.323	0.051		652.09	8.66	
9w (post day3)	Ctrl (n=21)	0.600	0.010	< 0.01	3.059	0.024	< 0.01	662.67	7.58	0.481
	Bmx/CA (n=23)	0.459	0.011		3.368	0.044		670.74	6.22	
10w	Ctrl (n=21)	0.622	0.011	< 0.01	3.023	0.027	< 0.01	687.52	6.53	0.418
	Bmx/CA (n=23)	0.495	0.014		3.349	0.044		681.52	5.27	
12w	Ctrl (n=21)	0.621	0.009	< 0.01	3.011	0.026	< 0.01	675.86	6.40	0.250
	Bmx/CA (n=23)	0.439	0.014		3.433	0.044		664.35	4.52	
16w	Ctrl (n=21)	0.599	0.009	< 0.01	3.122	0.040	< 0.01	678.71	5.59	< 0.05
	Bmx/CA (n=23)	0.415	0.013		3.587	0.046		658.83	7.40	
20w	Ctrl (n=21)	0.584	0.008	< 0.01	3.150	0.027	< 0.01	672.38	5.40	0.280
	Bmx/CA (n=23)	0.377	0.010		3.802	0.059		663.17	6.47	
24w	Ctrl (n=21)	0.596	0.010	< 0.01	3.163	0.032	< 0.01	668.05	8.57	0.331
	Bmx/CA (n=23)	0.365	0.010		4.040	0.094		662.27	6.60	
28w	Ctrl (n=21)	0.578	0.006	< 0.01	3.197	0.021	< 0.01	672.52	7.69	0.474
	Bmx/CA (n=23)	0.323	0.014		4.225	0.099		664.95	6.24	
32w	Ctrl (n=21)	0.581	0.009	< 0.01	3.177	0.044	< 0.01	658.33	10.00	0.395
	Bmx/CA (n=23)	0.293	0.012		4.464	0.133		653.09	6.63	
40w	Ctrl (n=3)	0.599	0.009	< 0.01	2.870	0.040	< 0.01	681.67	4.33	0.165
	Bmx/CA (n=3)	0.415	0.013		4.330	0.163		650.67	17.74	
48w	Ctrl (n=3)	0.584	0.008	< 0.01	2.990	0.020	< 0.01	496.33	18.85	< 0.05
	Bmx/CA (n=3)	0.377	0.010		5.333	0.283		564.00	14.15	
52w	Ctrl (n=2)	0.596	0.010	< 0.01	2.895	0.055	0.053	513.50	13.50	0.053
	Bmx/CA (n=5)	0.365	0.010		5.468	0.437		616.20	14.94	
56w	Ctrl (n=2)	0.578	0.006	< 0.01	2.840	0.030	0.083	646.50	31.50	0.564
	Bmx/CA (n=3)	0.323	0.014		5.787	0.712		632.00	18.18	
60w	Ctrl (n=5)	0.581	0.009	< 0.01	3.106	0.103	< 0.05	674.20	10.57	< 0.05
	Bmx/CA (n=4)	0.293	0.012		7.415	0.513		444.00	71.00	

Supplemental Table 2., Nakagawa et al.

Echocardiographic parameters of Ctrl and Bmx/CA mice treated with or without rNRG protein.

		LVFS				LVDd				HR			
		mean	SEM			mean	SEM			mean	SEM		
pre	Ctrl (n=15)	0.638	0.007	Ctrl vs Bmx/CA (*)	NS	2.917	0.032	*	NS	629.7	10.6	*	NS
	Bmx/CA (n=9)	0.618	0.011	Ctrl vs rNRG (§)	NS	2.944	0.059	§	NS	590.9	15.5	§	NS
	Bmx/CA + rNRG (n=9)	0.629	0.007	Bmx/CA vs rNRG (†)	NS	2.984	0.043	†	NS	630.1	16.7	†	NS
Tam d3	Ctrl (n=15)	0.642	0.006	*	P < 0.01	2.957	0.031	*	P < 0.01	654.3	10.0	*	NS
	Bmx/CA (n=9)	0.503	0.012	§	NS	3.190	0.045	§	NS	653.8	8.2	§	NS
	Bmx/CA + rNRG (n=9)	0.628	0.013	†	P < 0.01	3.041	0.048	†	NS	626.1	11.3	†	NS
Tam d5	Ctrl (n=15)	0.634	0.008	*	P < 0.01	2.961	0.026	*	P < 0.05	636.6	9.2	*	NS
	Bmx/CA (n=9)	0.459	0.015	§	NS	3.222	0.088	§	P < 0.05	638.7	7.6	§	P < 0.05
	Bmx/CA + rNRG (n=9)	0.626	0.013	†	P < 0.01	3.099	0.041	†	NS	600.8	9.3	†	P < 0.05
pTam d3	Ctrl (n=15)	0.643	0.007	*	P < 0.01	2.942	0.028	*	P < 0.01	675.1	5.1	*	NS
	Bmx/CA (n=9)	0.453	0.017	§	P < 0.05	3.373	0.073	§	NS	680.1	10.2	§	NS
	Bmx/CA + rNRG (n=9)	0.607	0.009	†	P < 0.01	3.080	0.049	†	P < 0.05	648.4	11.3	†	NS
pTam d6	Ctrl (n=15)	0.656	0.011	*	P < 0.01	2.876	0.032	*	P < 0.01	680.9	7.6	*	NS
	Bmx/CA (n=9)	0.428	0.016	§	P < 0.05	3.420	0.088	§	P < 0.01	649.6	32.5	§	P < 0.01
	Bmx/CA + rNRG (n=9)	0.607	0.014	†	P < 0.01	3.090	0.037	†	P < 0.01	634.7	10.9	†	NS
pTam d11	Ctrl (n=15)	0.638	0.009	*	P < 0.01	2.974	0.041	*	P < 0.01	696.1	6.0	*	NS
	Bmx/CA (n=9)	0.437	0.012	§	P < 0.05	3.523	0.080	§	P < 0.05	678.4	13.0	§	P < 0.01
	Bmx/CA + rNRG (n=9)	0.599	0.016	†	P < 0.01	3.220	0.062	†	P < 0.05	634.1	11.8	†	P < 0.05
pTam d14	Ctrl (n=15)	0.649	0.005	*	P < 0.01	2.946	0.048	*	P < 0.01	703.0	6.7	*	NS
	Bmx/CA (n=9)	0.421	0.007	§	NS	3.456	0.108	§	P < 0.01	706.7	12.0	§	P < 0.01
	Bmx/CA + rNRG (n=9)	0.604	0.020	†	P < 0.01	3.204	0.052	†	NS	639.0	7.7	†	P < 0.01
pTam d18	Ctrl (n=15)	0.633	0.008	*	P < 0.01	2.975	0.033	*	P < 0.01	704.2	5.6	*	NS
	Bmx/CA (n=9)	0.411	0.007	§	P < 0.05	3.447	0.083	§	P < 0.05	703.3	7.7	§	P < 0.01
	Bmx/CA + rNRG (n=9)	0.605	0.004	†	P < 0.01	3.213	0.085	†	NS	636.3	9.4	†	P < 0.01

Supplemental Table 3., Nakagawa et al.

Primer sequences and UPL numbers used for qRT-PCR.

Gene name	species	probe number	Fwd/Rev	sequences
Gapdh	mouse	80	Fwd	tgtccgtcgtggatctgac
			Rev	cctgcttcaccaccttcttg
Gapdh	human	63	Fwd	ccccggtttctataaattgagc
			Rev	caccttccccatggtgtct
Axin2	mouse	96	Fwd	gagagtgagcggcagagc
			Rev	cggctgactcgttctcct
Axin2	human	50	Fwd	ccacacccttctccaatcc
			Rev	tgccagtttctttggctctt
Lef1	mouse	94	Fwd	acccgtgatgggataaacag
			Rev	tcctgaaatccccaccttc
Nrg1	mouse	29	Fwd	tctgaccgaaggctctgc
			Rev	ggcctactgcaaaaccaaga
Nrg1	human	7	Fwd	aactgctgagggtgatggtc
			Rev	acttctcgtgacaccaccaa
Nppa	mouse	25	Fwd	cctcatcttctaccggcatc
			Rev	cacagatctgatggatttcaaga
Nppb	mouse	71	Fwd	agacccaggcagagtcagaa
			Rev	gtcagtcgtttgggctgtaac
Col1a1	mouse	15	Fwd	gcagctgacttcagggatg
			Rev	agacatgttcagctttgtggac
Col3a1	mouse	49	Fwd	tcccctggaatctgtgaatc
			Rev	tgagtcgaattggggagaat

Supplemental Figure S1., Nakagawa et al.

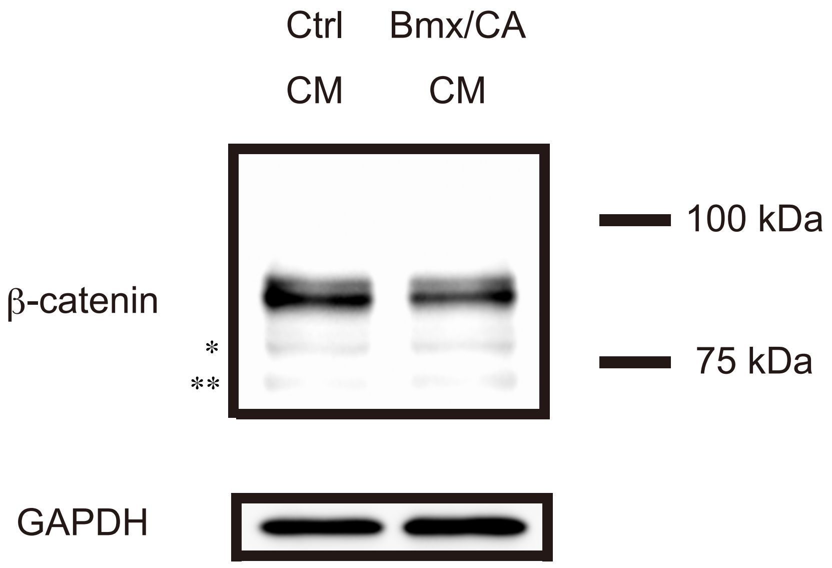

Supplemental Figure S1. β-catenin protein in the cardiomyocytes of Bmx/CA mice
Cardiomyocytes were isolated by langendorff perfusion apparatus and the expression of β-catenin protein was analyzed by western blotting. β-catenin protein lacking exon3 (= 75 kDa) was not detected in the cardiomyocytes from Ctrl and Bmx/CA mice. * and **, non-specific bands.

Supplemental Figure S2., Nakagawa et al.

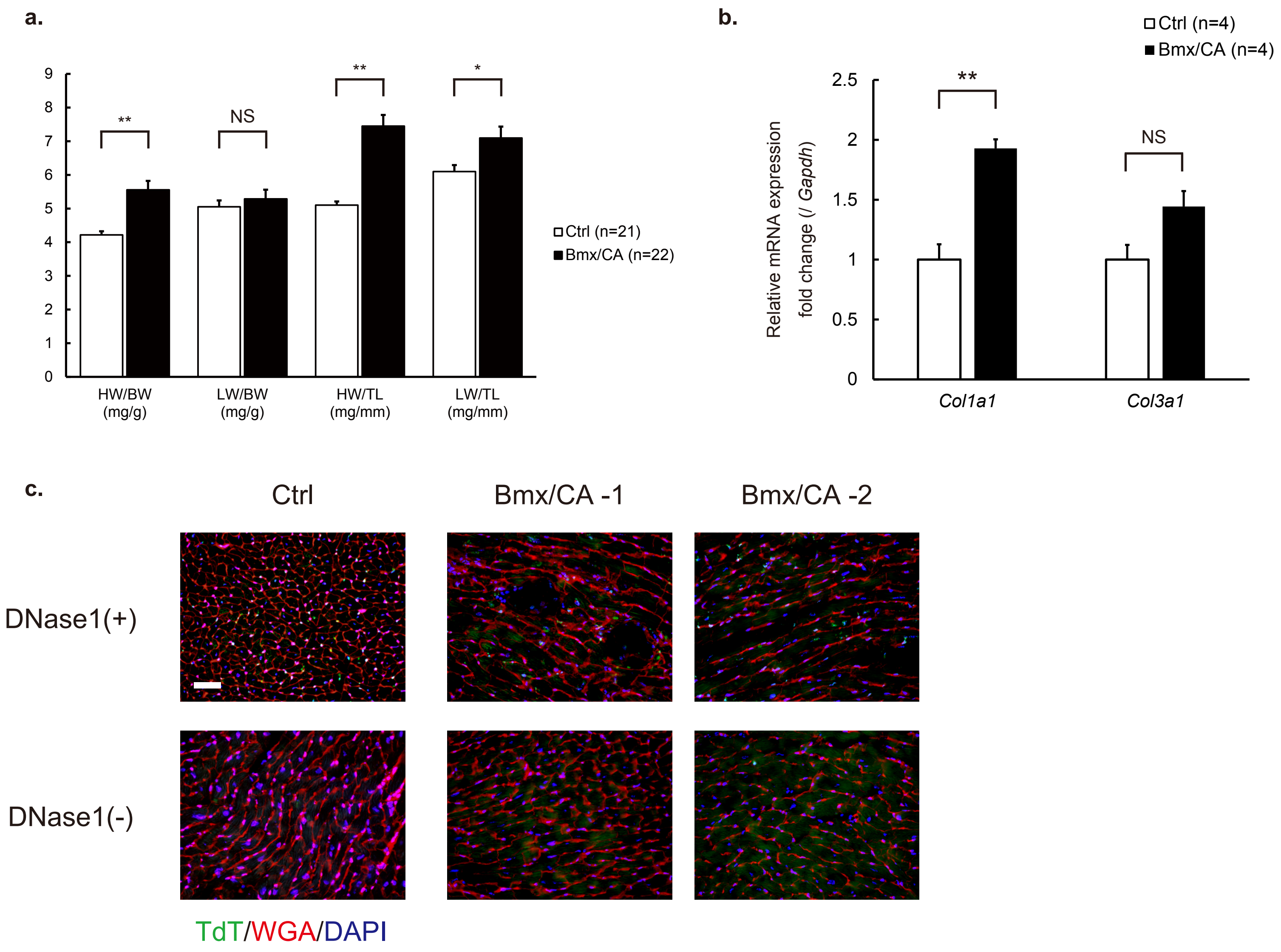

Supplemental Figure S2., Nakagawa et al. (continued)

d.

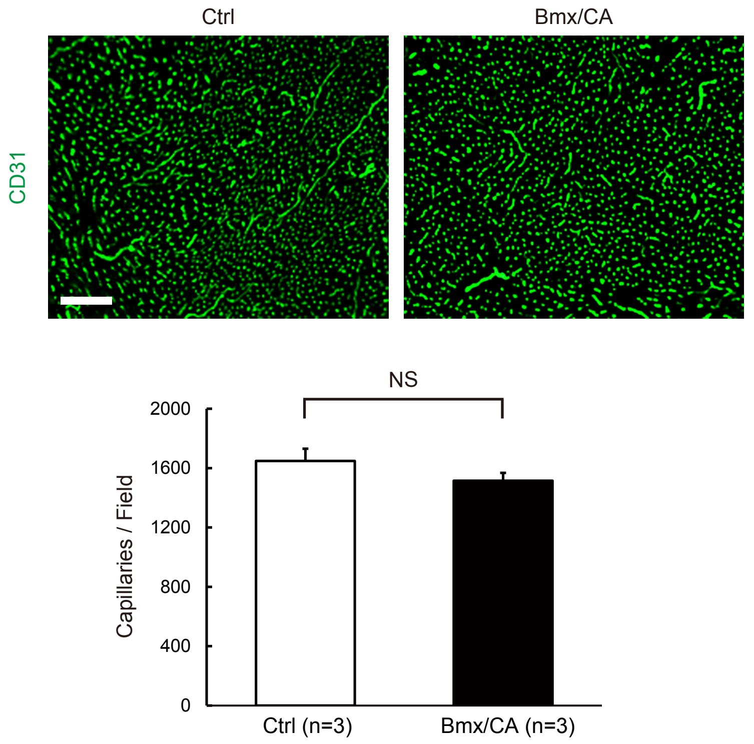

e.

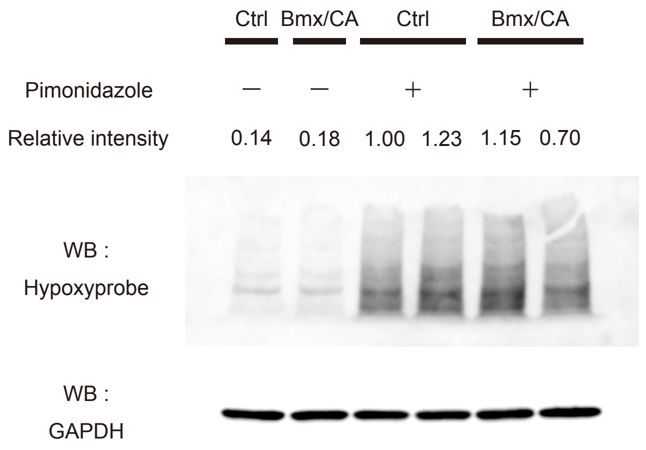

Supplemental Figure S2. Heart failure in Bmx/CA mice.
(a) Lung weight/Body weight ratio (LW/BW), Heart weight/Body weight ratio (HW/BW), Heart weight/Tibia length ratio (HW/TL) and Lung weight/Tibia length ratio (LW/TL) of Ctrl and Bmx/CA mice were examined at 6 months after TAM treatment. ** P<0.01, * P<0.05 versus Ctrl. (b) Quantitative RT-PCR analysis of the fibrosis related genes. Expression levels of Col1a1 and Col3a1 were examined at 6 months after TAM treatment. ** P<0.01 versus Ctrl. (c) TUNEL staining of heart tissues from Ctrl and Bmx/CA mice 6 months after TAM treatment. DNase1 were used for positive control. No staining was observed in the heart of both Ctrl and Bmx/CA mice. Scale bar: 50 μm. (d) Immunofluorescent staining of heart tissues for CD31 (green). The capillary vessels in Ctrl and Bmx/CA hearts were counted on 10 randomly selected fields (533 × 667 μm2). Scale bar: 100 μm. (e) Pimonidazole binds to thiol-containing proteins specifically in hypoxic cells. Tissue hypoxia was assessed by calculating the band intensity of anti-pimonidazole antibody reacting protein. Band density was calculated using ImageJ.
