# Supplementary material for: Author Correction: Activation of endothelial β-catenin signaling induces heart failure
Source: Sci Rep. 2018 Oct 29;8:15858. doi: 10.1038/s41598-018-34062-x (PMC6206138; doi:10.1038/s41598-018-34062-x)
Supplement: Supplementary file 1 — Supplementary Table S1 [file 41598_2018_34062_MOESM1_ESM.pdf]

Corrected Supplemental Table 1., Nakagawa et al.

Echocardiographic parameters of Ctrl and Bmx/CA mice after tamoxifen treatment.

		LVFS			LVDd (mm)			HR (bpm)		
		mean	SEM	p-value	mean	SEM	p-value	mean	SEM	p-value
pre	Ctrl (n=21)	0.599	0.013	0.698	2.964	0.032	0.084	666.52	11.65	< 0.01
	Bmx/CA (n=23)	0.604	0.012		3.041	0.029		641.17	7.87	
8w (Tam3)	Ctrl (n=21)	0.600	0.008	< 0.01	2.920	0.038	< 0.01	674.00	6.44	0.860
	Bmx/CA (n=23)	0.514	0.011		3.110	0.031		670.13	7.11	
8w (Tam5)	Ctrl (n=21)	0.599	0.010	< 0.01	3.026	0.039	< 0.01	647.81	7.37	0.805
	Bmx/CA (n=23)	0.462	0.013		3.323	0.051		652.09	8.66	
9w (post day3)	Ctrl (n=21)	0.600	0.010	< 0.01	3.059	0.024	< 0.01	662.67	7.58	0.481
	Bmx/CA (n=23)	0.459	0.011		3.368	0.044		670.74	6.22	
10w	Ctrl (n=21)	0.622	0.011	< 0.01	3.023	0.027	< 0.01	687.52	6.53	0.418
	Bmx/CA (n=23)	0.495	0.014		3.349	0.044		681.52	5.27	
12w	Ctrl (n=21)	0.621	0.009	< 0.01	3.011	0.026	< 0.01	675.86	6.40	0.250
	Bmx/CA (n=23)	0.439	0.014		3.433	0.044		664.35	4.52	
16w	Ctrl (n=21)	0.599	0.009	< 0.01	3.122	0.040	< 0.01	678.71	5.59	< 0.05
	Bmx/CA (n=23)	0.415	0.013		3.587	0.046		658.83	7.40	
20w	Ctrl (n=21)	0.584	0.008	< 0.01	3.150	0.027	< 0.01	672.38	5.40	0.280
	Bmx/CA (n=23)	0.377	0.010		3.802	0.059		663.17	6.47	
24w	Ctrl (n=21)	0.596	0.010	< 0.01	3.163	0.032	< 0.01	668.05	8.57	0.331
	Bmx/CA (n=23)	0.365	0.010		4.040	0.094		662.27	6.60	
28w	Ctrl (n=21)	0.578	0.006	< 0.01	3.197	0.021	< 0.01	672.52	7.69	0.474
	Bmx/CA (n=23)	0.323	0.014		4.225	0.099		664.95	6.24	
32w	Ctrl (n=21)	0.581	0.009	< 0.01	3.177	0.044	< 0.01	658.33	10.00	0.395
	Bmx/CA (n=23)	0.293	0.012		4.464	0.133		653.09	6.63	
40w	Ctrl (n=3)	0.618	0.012	< 0.01	2.870	0.040	< 0.01	681.67	4.33	0.165
	Bmx/CA (n=3)	0.276	0.027		4.330	0.163		650.67	17.74	
48w	Ctrl (n=3)	0.619	0.024	< 0.01	2.990	0.020	< 0.01	496.33	18.85	< 0.05
	Bmx/CA (n=3)	0.209	0.020		5.333	0.283		564.00	14.15	
52w	Ctrl (n=2)	0.629	0.026	< 0.01	2.895	0.055	0.053	513.50	13.50	0.053
	Bmx/CA (n=5)	0.167	0.023		5.468	0.437		616.20	14.94	
60w	Ctrl (n=5)	0.628	0.014	< 0.01	3.106	0.103	< 0.05	674.20	10.57	< 0.05
	Bmx/CA (n=4)	0.109	0.005		7.415	0.513		444.00	71.00	
